# Cardiovascular magnetic resonance-determined left ventricular myocardium impairment is associated with C-reactive protein and ST2 in patients with paroxysmal atrial fibrillation

**DOI:** 10.1186/s12968-021-00732-5

**Published:** 2021-03-22

**Authors:** Lei Zhao, Songnan Li, Chen Zhang, Jie Tian, Aijia Lu, Rong Bai, Jing An, Andreas Greiser, Jie Huang, Xiaohai Ma

**Affiliations:** 1 Department of Radiology, Beijing Anzhen Hospital, Capital Medical University, Beijing, China; 2 Department of Cardiology, Beijing Anzhen Hospital, Capital Medical University, Beijing, China; 3 Siemens Shenzhen Magnetic Resonance Ltd, Shenzhen, China; 4 Siemens AG Healthcare, Erlangen, Germany; 5 Department of Radiology, Michigan State University, East Lansing, USA; 6 Department of Intervention, Beijing Anzhen Hospital, Capital Medical University, Beijing, China

**Keywords:** Atrial fibrillation, Left ventricle, Myocardial strain, T1 mapping, Biomarkers

## Abstract

**Background:**

Myocardial strain assessed with cardiovascular magnetic resonance (CMR) feature tracking can detect early left ventricular (LV) myocardial deformation quantitatively in patients with a variety of cardiovascular diseases, but this method has not yet been applied to quantify myocardial strain in patients with atrial fibrillation (AF) and no coexistent cardiovascular disease, i.e., the early stage of AF. This study sought to compare LV myocardial strain and T1 mapping indices in AF patients and healthy subjects, and to investigate the associations of a portfolio of inflammation, cardiac remodeling and fibrosis biomarkers with LV myocardial strain and T1 mapping indices in AF patients with no coexistent cardiovascular disease.

**Methods:**

The study consisted of 80 patients with paroxysmal AF patients and no coexistent cardiovascular disease and 20 age- and sex-matched healthy controls. Left atrial volume (LAV), LV myocardial strain and native T1 were assessed with CMR, and compared between the AF patients and healthy subjects. Biomarkers of C-reactive protein (CRP), transforming growth factor beta-1 (TGF-β1), collagen III N-terminal propeptide (PIIINP), and soluble suppression of tumorigenicity 2 (sST2) were obtained with blood tests, and compared between the AF patients and healthy controls. Associations of these biomarkers with those CMR-measured parameters were analyzed for the AF patients.

**Results:**

For the CMR-measured parameters, the AF patients showed significantly larger LAV and LV end-systolic volume, and higher native T1 than the healthy controls (max P = 0.027). The absolute values of the LV peak systolic circumferential strain and its rate as well as the LV diastolic circumferential strain rate were all significantly reduced in the AF patients (all P < 0.001). For the biomarkers, the AF patients showed significantly larger CRP (an inflammation biomarker) and sST2 (a myocardium stiffness biomarker) than the controls (max P = 0.007). In the AF patients, the five CMR-measured parameters of LAV, three LV strain indices and native T1 were all significantly associated with these two biomarkers of CRP and sST2 (max P = 0.020).

**Conclusions:**

In patients with paroxysmal AF and no coexistent cardiovascular disease, LAV enlargement and LV myocardium abnormalities were detected by CMR, and these abnormalities were associated with biomarkers that reflect inflammation and myocardial stiffness.

**Supplementary Information:**

The online version contains supplementary material available at 10.1186/s12968-021-00732-5.

## Background

Atrial fibrillation (AF) is the most common sustained arrhythmia. The estimated number of AF patients was 33.5 million worldwide in 2010, and this number is increasing, presenting a rapidly growing public health burden [1]. AF is associated with structural, electrical and contractile remodeling of the atria. In addition, AF is also associated with systemic inflammation, diffuse fibrosis and adverse effects on the left ventricular (LV) myocardium [2]. Emerging evidence demonstrates that myocardial strain, fibrosis and inflammation are associated with AF as well as the pathogenesis of the arrhythmia [3]. Circulating biomarkers of C-reactive protein (CRP), transforming growth factor beta-1 (TGF-β1), collagen III N-terminal propeptide (PIIINP) and suppression of tumorigenicity 2 (ST2) are found to be linked with AF, and these biomarkers may reflect inflammatory or profibrotic processes or in response to pressure or volume overload [4–7]. In a large East Asians population from a health screening program, Kwon et al. found that the elevation of serum CRP is associated with the prevalence and risk of AF, which suggest that inflammation plays a role in the pathogenesis of AF [4]. Higher levels of fibrotic biomarkers such as TGF-β1 and PIIINP are found to be associated with extensive myocardial fibrosis, especially atrial fibrosis, and may act as predictors for adverse outcomes such as AF recurrences after ablation or post-operative AF [2, 5, 6]. As a biomarker of cardiomyocyte stretch, sST2 is found to be higher in AF patients and also predicts LV dysfunction [7]. And these biomarkers are reported to be associated with new onsets of AF [8–11]. These elevated circulating biomarkers may reflect myocardial abnormalities in AF, both in atrium and ventricle, and the latter usually represents the abnormalities of combined cardiovascular diseases. In AF, up to one-third of cases occur in the absence of apparent cardiovascular disease [2]. In contrast to the advanced disease condition which has been investigated extensively, only subtle LV abnormalities are reported in AF with no coexistent cardiovascular disease [12–14]. It is unclear whether these LV abnormalities are related to circulating biomarkers that reflect several pathological conditions.

Myocardial strain assessed with cardiovascular magnetic resonance (CMR) feature tracking can detect early LV myocardial deformation quantitatively in patients with a variety of cardiovascular diseases, and T1 mapping can detect LV myocardial abnormalities and is found to be correlated well with myocardial fibrosis in patients with AF and combined cardiovascular diseases [15, 16]. However, to the best of our knowledge, the application of CMR methods to quantify myocardial strain and T1 value for assessing LV myocardium abnormalities in AF patients with no coexistent cardiovascular disease has not been reported. In this study, we sought to compare LV myocardial strain indices and native T1 between an AF population and healthy controls, and to investigate associations between the circulating biomarkers and CMR LV quantitative indices for the AF population.

## Methods

### Study population

**Fig. 1:**
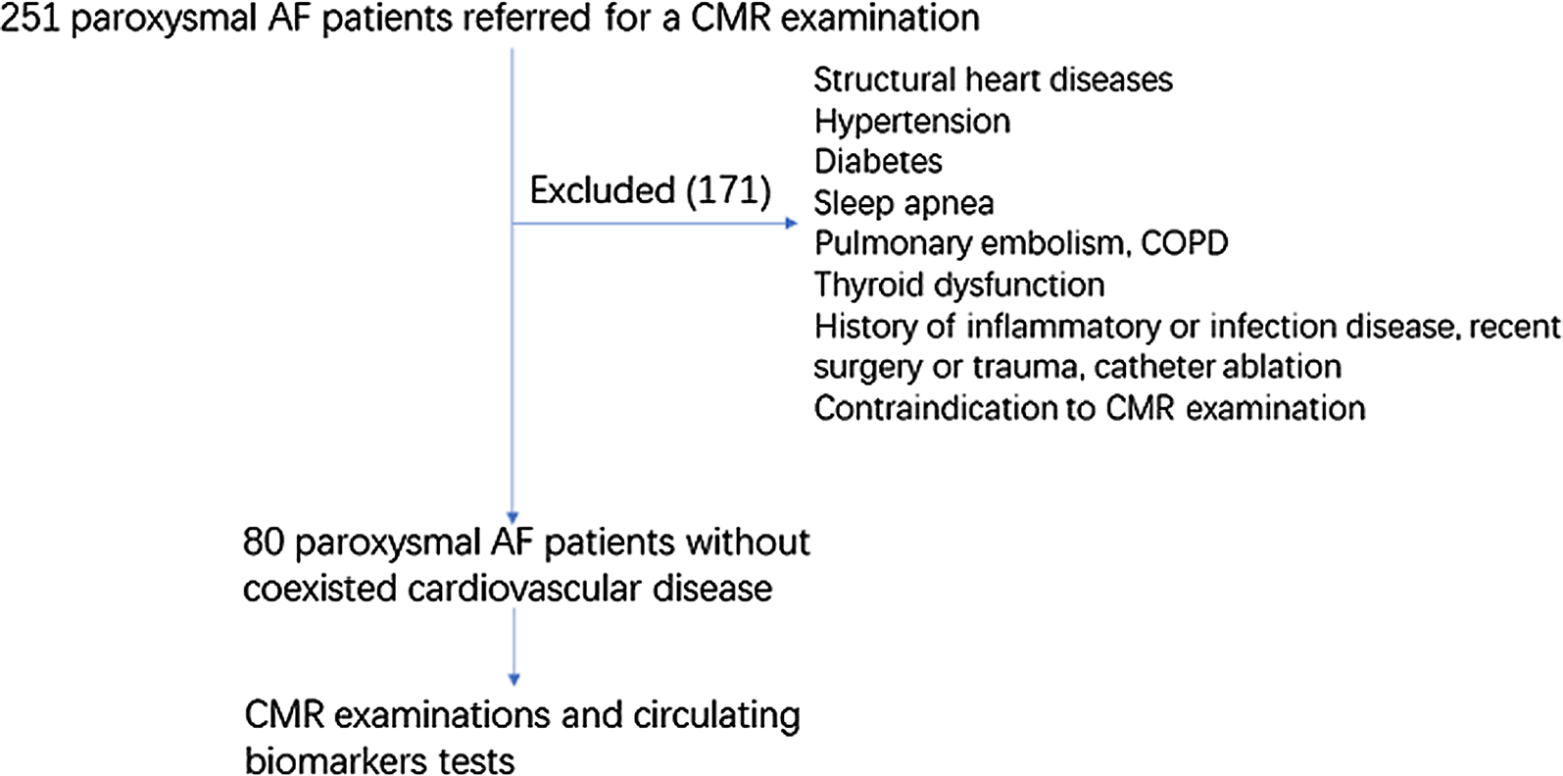
Flow chart of the study. According to the criteria, we enrolled 80 paroxysmal AF patients between November 2015 and December 2016. *CMR* cardiovascular magnetic resonance. *COPD* chronic obstructive pulmonary disease

We performed a prospective observational study of patients with paroxysmal AF and no coexistent cardiovascular disease. All patients were diagnosed with paroxysmal AF in accordance with the contemporary clinical guidelines [17]. Exclusion criteria included any significant structural heart diseases, hypertension, diabetes, sleep apnea, pulmonary embolism, chronic obstructive pulmonary disease, thyroid dysfunction, history of inflammatory or infection disease, recent surgery or trauma (within the last 4 weeks), prior catheter ablation, and contraindication to CMR. With these criteria, we enrolled 80 AF patients between November 2016 and December 2017. They underwent clinical assessment, CMR examination, and biomarker tests (Fig. 1). 20 age- and sex-matched subjects without any history of cardiovascular diseases were recruited as healthy controls. They underwent CMR examination and biomarker tests to provide reference values. The study was approved by our Institutional Review Board, and written informed consents were obtained from all subjects.

### CMR examinations

**Fig. 2:**
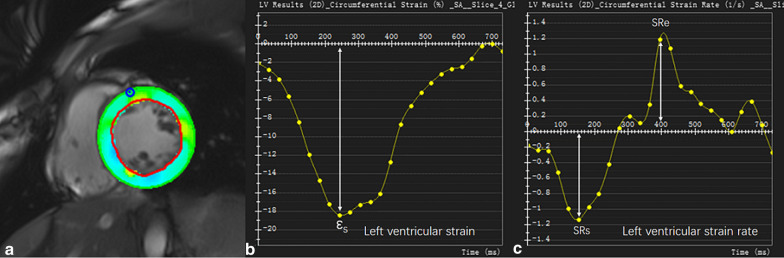
CMR strain images. Illustration of left ventricular (LV) tracking on mid-ventricular level short-axis cine images in a patient with paroxysmal atrial fibrillation (AF) patient. **a** Mid-ventricular level short-axis cine image. Borders of endocardial (red line) and epicardial borders (green line) were manually delineated with the initial contour set at end-diastole. Papillary muscles were excluded from the endocardial contour. **b** The LV 2D circumferential strain curve. The peak systolic circumferential strain (ε_s_) was identified from the strain curve. **c** The LV strain rate curve. The peak systolic circumferential strain rate (SRs) and early diastolic circumferential strain rate (SRe) were determined from the strain rate curve

All CMR examinations were performed using a 3T CMR system (MAGNETOM Verio, Siemens Healthineers, Erlangen, Germany) with a 32-channel cardiac coil. Balanced steady-state free-precession cine images were obtained during repeated breath-holds in two long axes (horizontal and vertical) and in a stack of short-axis slices covering the LV. Imaging parameters were: repetition time (TR) = 3.1 ms, echo time (TE) = 1.3 ms, flip angle (FA) = 45°, field of view (FOV) = 276 × 340 mm^2^, matrix = 156 × 192, slice thickness = 6 mm, receiver bandwidth (BW) = 977 Hz/px, parallel imaging using GRAPPA reconstruction (R = 2), and 25 cardiac phases. T1 mapping was performed using the modified look-locker inversion recovery sequence [MOLLI, sampling pattern: 5(3)3]. Data were acquired in the mid-ventricular level short-axis plane before contrast administration (Fig. 2). Imaging parameters were: TR = 2.6–2.7 ms, TE = 1.0–1.1 ms, FA = 35°, FOV = 270 × 360 mm^2^, matrix = 256 × 256, slice thickness = 6 mm, BW = 1045−1028 Hz/px, GRAPPA acceleration factor 2, and linear phase-encoding ordering. Quality control was performed during scanning by monitoring the “goodness of fit” map and source images to allow an immediate repetition of suboptimal measurements to minimize the respiratory motion and off-resonance effects. 3 patients encountered arrhythmia during the scan, and the scan was repeated later under the sinus rhythm for each patient. Late gadolinium enhancement (LGE) imaging was performed in the same planes as cine images using a segmented inversion-recovery gradient-echo sequence 15 min after administration of 0.1 mmol/kg gadopentetate dimeglumine (Magnevist, Bayer Healthcare, Berlin, Germany). The inversion time was repeatedly adjusted to appropriately null the myocardium during the length of LGE image acquisition. Imaging parameters were: TR = 4.1 ms, TE = 1.6 ms, FA = 20°, FOV = 350 × 262 mm^2^, matrix = 256 × 162, slice thickness = 6 mm, BW = 461 Hz/px, and GRAPPA acceleration factor 2.

The global LV functional indices were analyzed using dedicated software (Argus, Siemens Healthineers). The following indices were measured: LV ejection fraction (LVEF), LV end-diastolic volume (LVEDV), LV end-systolic volume (LVESV), LV stroke volume (SV), LV cardiac output (CO), and LV mass. Left atrium volume (LAV) was measured at end-systole using the biplane area-length method. LGE images were examined to identify any replacement fibrosis within the LV myocardium. A replacement fibrosis was considered present only if it was confirmed on both short-axis and matching long-axis myocardial locations. If a positive lesion was found, further quantitative assessment would be performed. All the same mid-ventricular level short-axis cine images (for strain analysis) and T1 mapping images were analyzed. The strain analysis was performed with custom software (cvi42, Calgary, Alberga, Canada) (Fig. 2). LV endocardial and epicardial borders were manually delineated with the initial contour set at end-diastole. Papillary muscles were excluded from the endocardial contour. LV peak systolic circumferential strain (ε_s_), peak systolic circumferential strain rate (SRs), and early diastolic circumferential strain rate (SRe) were obtained from cine images for both patients and healthy controls. The T1 maps and source images of all subjects were assessed, and myocardial segments with artifacts were excluded for further analysis. The LV myocardium was delineated by manually contouring the endocardial and epicardial borders. Care was taken to avoid contamination of signal from blood or epicardial fat. The global T1 of mid-ventricular level LV myocardium for each patient or healthy control was obtained.

### Biomarkers analysis

Venous blood samples were collected from the antecubital vein in the morning before the CMR examination. Plasma high-sensitivity CRP (hs-CRP) concentration was determined by standard quantitative assay techniques in our Department of Clinical Laboratory according to the manufacturer’s protocol. The measurement range and detection limit of this test were 0.25–10.00 mg/L and 0.25 mg/L, respectively. The serum level of TGF-β1 was determined with an enzyme-linked immunosorbent assay (ELISA) using a commercially available kit (Yihan International, Inc, Shanghai, China). The kits were processed according to the manufacturer’s instructions. PIIINP detection was determined with a commercially available radio-immuno-assay (Orion Diagnostica, Espoo, Finland). Soluble ST2 (sST2) was analyzed using a high-sensitivity Presage ST2 assay (Critical Diagnostics, San Diego, California, USA). The measurement range of the sST2 assay was 3.1 to 200 ng/mL with an intra-assay coefficient of variation (CV) % of 5.1% and inter-assay CV% of 5.2%. All samples with a level of sST2 more than 200 ng/mL were diluted further to provide quantitative results according to the manufacturer’s protocol.

The intra- and inter-observer variability for the CMR parameters were assessed by the intraclass correlation coefficient (ICC) of the CMR parameters measured for 50 randomly selected subjects (40 AF patients and 10 healthy controls). A same observer re-measured these parameters, and the intra-observer reproducibility was determined with the ICC of the second measures with the first measures. A second-independent observer, blinded to the first observer’s measures, measured these parameters, and the inter-observer reproducibility was assessed with the ICC of these two different observers’ measures.

### Statistical analysis

Continuous data are presented as mean ± standard deviation (SD) or median [interquartile range (IQR), 25th–5th percentile] and categorical variables as a percentage. Owing to the skewed distribution of biomarkers, analyses were performed after log transformation. Continuous data were compared using an unpaired Student t-test or Mann–Whitney nonparametric U test as appropriate. Nominal data are presented as numbers and percentages and were compared with the Chi-square test. We analyzed the associations of LV strain indices and native T1 time with the circulating biomarkers using the Pearson correlation analysis. A two-sided P value < 0.05 was considered statistically significant. All statistical analyses were performed using SPSS software (version 21, Statistical Package for the Social Sciences, International Business Machines, Inc., Armonk, New York, USA ).

## Results

The patients with paroxysmal AF had a mean age of 50 ± 10 years with 88.8% males. The clinical characteristics of these patients and the healthy controls are presented in Table 1. These patients with AF showed a significantly higher heart rate (P < 0.001). Both systolic and diastolic blood pressures were similar between groups. Circulating biomarkers of the AF patients and healthy controls are presented in Table 2. There was no significant difference between groups for both TGF-β1 and PIIINP, but levels of hs-CRP and sST2 were significantly higher in the AF patients (both P < 0.01).

**Table 1:** Clinical characteristics of the study population with atrial fibrillation (AF) and healthy controls

Variables	Paroxysmal AF patients (n = 80)	Healthy controls (n = 20)	P-value
Age, years	50 ± 10	50 ± 5	0.807
Male (%)	71 (88.8)	17 (85.0)	0.644
Time from the first diagnosis, months (median, IQR)	23, 6–47	–	–
Systolic BP, mmHg	124 ± 11	119 ± 12	0.117
Diastolic BP, mmHg	80 ± 10	78 ± 9	0.427
AF burden, min/day (median, IQR)^a^	35, 15–89	–	–
Smoking (%)	33 (41.3)	8 (40.0)	0.919
BMI, kg/m^2^	26.8 ± 3.2	26.7 ± 1.6	0.829
Heart rate, beats/min	72 ± 12	63 ± 2	< 0.001

Data are expressed as mean ± SD or number (%) or median, IQR

*BP* blood pressure, *AF* atrial fibrillation, *BMI* body mass index

^a^Each patient enrolled in this study had at least one 24-h Holter monitoring. The AF burden was calculated with the recorded episodes of AF for 31 AF patients with reported AF during the Holter monitoring

**Table 2:** Circulating biomarkers of the study population with paroxysmal AF and healthy controls

Variables	Paroxysmal AF patients (n = 80)	Healthy controls (n = 20)	P-value
hs-CRP, mg/L	2.2 (1.7–2.3)	1.5 (0.6–1.6)	< 0.001
sST2, ng/mL	16.5 (14.1–18.5)	13.6 (8.5–14.9)	0.007
TGF-β1, ng/mL	12.9 (8.5–15.9)	11.2 (8.3–13.6)	0.480
PIIINP, µg/L	5.5 (3.4–9.9)	4.0 (3.0-10.4)	0.965

Data are expressed as median (IQR)

*hs-CRP* high-sensitivity C-reactive protein, *sST2* soluble suppression of tumorigenicity 2, *TGF-β1* transforming growth factor beta-1; *PIIINP* collagen III N-terminal propeptide

CMR-measured LAV and LV parameters for both AF patients and healthy controls are presented in Table 3. Compared to the healthy controls, the AF patients showed a significantly larger LAV (P < 0.001). The LVEDV of the AF patients was similar as that of the healthy controls, but the LVESV of the AF larger (P = 0.027) and a trend for the LVEF of AF patients to be reduced (P = 0.051). In comparison, both LV SV and LV mass were similar. The absolute values of all three LV strain indices were significantly reduced for the AF cohort (all P < 0.001). The LGE examination did not detect LV replacement fibrosis in either AF patient or healthy controls. One patient showed a linear high signal intensity in the mid-wall of the basal septal segment at the first short-axis image, and, after carefully reviewing different orientations of LGE images with matched cine images, it indicated a membranous interventricular septum. The LV native T1 times were significantly higher for the AF patients (P < 0.001).

**Table 3:** CMR characteristics of the study population with AF and controls

Variables	AF patients (n = 80)	Healthy controls (n = 20)	P-value
LAV, mL	122 ± 32	103 ± 11	< 0.001
LVEF, %	62 ± 9	66 ± 7	0.051
LVEDV, mL	123 ± 26	120 ± 27	0.708
LVESV, mL	47 ± 12	41 ± 13	0.027
LV SV, mL	76 ± 16	79 ± 18	0.594
LV mass, g	130 ± 35	122 ± 33	0.118
LV peak SCS (%)	− 18.2 ± 2.0	− 21.9 ± 1.1	< 0.001
LV peak SCS rate (s^− 1^)	− 1.15 ± 0.16	− 1.21 ± 0.03	< 0.001
LV early DCS rate (s^− 1^)	1.20 ± 0.18	1.35 ± 0.13	< 0.001
LV LGE	0	–	–
LV native T1 time, ms	1280 ± 33	1251 ± 12	< 0.001

Data are expressed as mean ± SD

*CMR* cardiovascular magnetic resonance, *AF* atrial fibrillation, *LV* left ventricle/left ventricular, *LAV* left atrium volume, *LVEF* left ventricular ejection fraction, *LVEDV* left ventricular end-diastolic volume, *LVESV* left ventricular end-systolic volume, *SV* stroke volume, *SCS* systolic circumferential strain, *DCS* diastolic circumferential strain, *LGE* late gadolinium enhancement

**Fig. 3:**
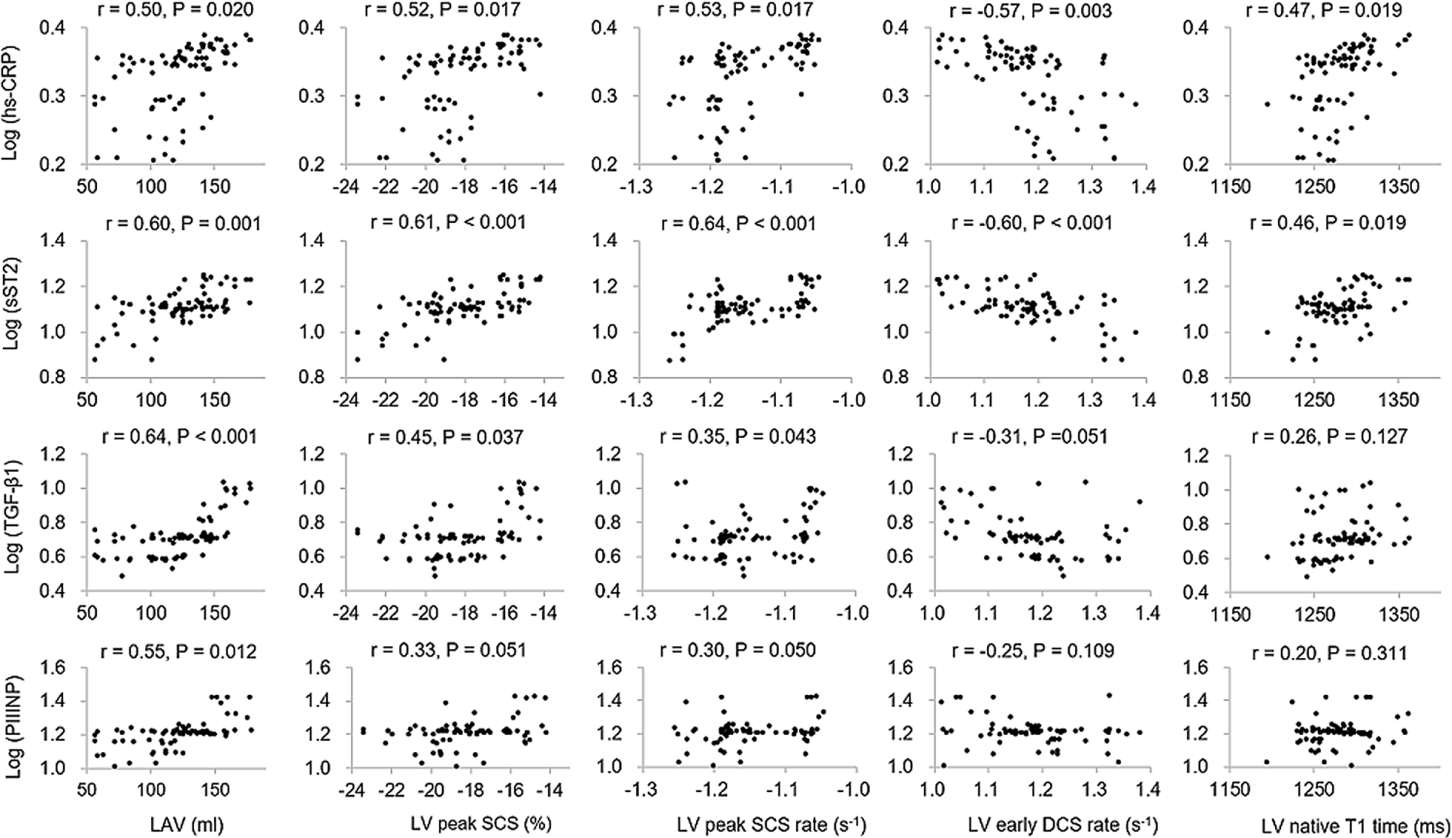
Scatter plots of the correlations of the five CMR-measured parameters with the four tested circulating biomarkers in the AF patients. The Y axis of the five plots in the top row is log (hs-CRP), the second row is log (sST2), the third row log (TGF-β1), and the bottom row log (PIIINP), respectively. The X axis of the four plots in the left column is LAV, the second column is LV peak SCS, the third column plots LV peak SCS rate, the fourth column LV early DCS rate, and the right column LV native T1 time, respectively. *LAV* left atrium volume, *LV* left ventricle, *SCS* systolic circumferential strain, *DCS* diastolic circumferential strain, *hs-CRP* high-sensitivity C-reactive protein, *sST2* soluble suppression of tumorigenicity 2, *TGF-β1* transforming growth factor beta-1, *PIIINP* collagen III N-terminal propeptide

For AF patients, the results of the correlation analysis of all five CMR-measured parameters with all four circulating biomarkers are tabulated in Table 4, and their corresponding scatter plots are illustrated in Fig. 3. LAV was significantly associated with all four circulating biomarkers of hs-CRP, sST2, TGF-β1 and PIIINP (max P = 0.020). Both LV peak systolic circumferential strain and strain rate were positively correlated with hs-CRP, sST2 and TGF-β1 (max P = 0.043), respectively. LV early diastolic circumferential strain rate was negatively correlated with hs-CRP and sST2 (max P = 0.001). LV native T1 times were significantly and positively correlated with hs-CRP and sST2 (max P = 0.019), though the strength of this correlation is relatively weak.

**Table 4:** Correlations of LAV, LV strain indices and native T1 with circulating biomarkers in the AF patients

	hs-CRP			sST2		TGF-β1			PIIINP	
	r		P	r	P	r	P		r	P
LAV	0.50		0.020	0.60	0.001	0.64	< 0.001		0.55	0.012
LV peak SCS	0.52		0.017	0.61	< 0.001	0.45	0.037		0.33	0.051
LV peak SCS rate	0.53		0.017	0.64	< 0.001	0.35	0.043		0.30	0.050
LV early DCS rate	− 0.57		0.003	− 0.60	< 0.001	− 0.31	0.051		− 0.25	0.109
LV native T1 time	0.47		0.019	0.46	0.019	0.26	0.127		0.20	0.311

*LAV* left atrial volume, *SCS* systolic circumferential strain, *DCS* diastolic circumferential strain, *hs-CRP* high-sensitivity C-reactive protein, *sST2* soluble suppression of tumorigenicity 2, *TGF-β1* transforming growth factor beta-1, *PIIINP* collagen III N-terminal propeptide

The ten CMR-measured parameters were highly reproducible on an intra- and inter-observer level, reflected in the mean ICC 0.92 (± 0.03) for the intra-observer reproducibility and 0.88 (± 0.04) for the inter-observer reproducibility, respectively. The ICC values for both intra- and inter-observer reproducibility are tabulated in Additional file 1: Table S1.

## Discussion

AF is the most common sustained arrhythmia. In the present study of patients with paroxysmal non-valvular AF and no coexistent cardiovascular disease, we evaluated LAV and LV function with CMR cine image analysis, and LV tissue characterization with CMR native T1 mapping. AF patients had significantly increased LAV (Table 3). Impaired LV function was demonstrated with the significantly reduced absolute values of all three LV circumferential strain indices (Table 3). The AF patients also showed a small but significant increase in native T1 (Table 3), demonstrating a potential LV myocardial abnormality in these AF patients. This myocardial abnormality may affect the LV function. These results provide evidence to show abnormalities of both the LA and LV in paroxysmal AF patients with no apparent cardiovascular disease.

AF without any underlying cardiovascular disease is thought to be an early stage of the disease and occurs in about one third of the AF population [13, 18], but sparse data report the ventricular abnormality in this population [12, 13]. Myocardial strain imaging has shown to be able to detect early contractile dysfunction in many cardiovascular diseases [19]. For the early stage of AF, this method may detect myocardial subtle changes before any noticeable presence of significant morphological abnormalities. Conventional LV functional indices mainly indicate the systolic function. In contrast, the strain method includes indices that reflect both systolic and diastolic function. With CMR strain method, our data showed that although the absolute values of the LV peak systolic circumferential strain, peak systolic circumferential SR, and early diastolic circumferential SR were significantly reduced in the AF patients (all P < 0.001, Table 3), the LVEF of the AF patients was only marginally reduced compared to the healthy controls (P = 0.051), showing that this CMR-measured LV dysfunction reflected an early stage of AF in the patients with paroxysmal AF and no coexistent cardiovascular disease.

The present study also measured the AF-associated circulating biomarkers and investigated the association of those CMR-measured parameters with these circulating biomarkers. The AF patients showed similar levels of TGF-β1 and PIIINP as that of the healthy controls, but significantly elevated hs-CRP and sST2 (Table 2), indicating that both hs-CRP and sST2 may be associated with the impairment of both LA and LV. Indeed, all the five CMR-measured parameters of LAV, three LV circumferential strain indices and LV native T1 times were significantly associated with both hs-CRP and sST2 (Table 4). These results show a role of these two circulating biomarkers in those abnormalities of both LV and LV in the patients with paroxysmal AF and no coexistent cardiovascular disease.

Previous studies report elevated levels of these circulating biomarkers that reflect inflammation, cardiac remodeling and fibrosis biomarkers in AF patients coexisting with hypertension, diabetes, heart failure and myocardial infarction [20–24]. Inflammation is found to be involved in the development and perpetuation of AF and AF-related thrombosis [23]. It also participates in the process of cardiac remodeling that leads to interstitial myocardial fibrosis [25]. The inflammation biomarker of CRP is found to be elevated in AF patients without coexistent cardiovascular disease [26]. The relationships between CRP and myocardial strain indices are reported in many cardiovascular diseases [27–30], and systemic inflammation is found to be linked to the impairment of myocardial contractility [29]. CRP is also reported to be associated with LV T1 mapping indices in patients with systemic inflammation involving the heart or general population without known cardiovascular disease [25, 31]. The present study found a significantly elevated CRP in the AF patients (Table 2), and significant associations between the CRP and all five CMR-measured parameters of LAV, three LV circumferential strain indices and LV native T1 (Table 4), showing that at the early stage of AF inflammation might play a role in impairing morphology and function of both LA and LV in the paroxysmal AF patients with no coexistent cardiovascular disease. Anti-inflammation therapies may be considered as a management to AF, and the CMR can be used to evaluate the effectiveness of these therapies. However, the underlying mechanisms that link inflammation with abnormal LAV, LV function and myocardium impairments remain unclear. Future investigations with intervention and longitudinal observations are needed to provide insight into the relationship between inflammation and LV function and myocardium impairments in AF patients.

The biomarker sST2 reflects myocardium stiffness and its concentration rises in response to myocardial strain [32]. The biology of the ST2 system is complex, and ST2 is a member of the Toll-like/interleukin-1 receptor superfamily [33]. Soluble ST2 mediates myocardial strain and plays a role in cardiac remodeling [33]. sST2 is suggested to be associated with new onsets of AF, but not correlated with the amount of cardiac fibrosis and affected by inflammation process [8, 11, 34]. Our study also found a significantly elevated sST2 in the AF patients (Table 2), and significant associations between this biomarker and all five CMR-measured parameters of LAV, three LV circumferential strain indices and LV native T1 times (Table 4), showing that at the early stage of AF this elevated sST2 might also play an important role in impairing morphology and function of both LA and LV in the paroxysmal AF patients and no coexistent cardiovascular disease.

LV abnormalities, especially fibrotic changes, are identified with imaging methods in AF patients, and more extensive changes are found in persistent AF than paroxysmal AF [12, 15, 35]. AF patients in these studies usually coexist with risk factors such as hypertension, diabetes, coronary artery disease, etc., which contribute to ventricular fibrosis. The two fibrotic biomarkers of TGF-β1 and PIIINP are found to be associated with extensive myocardial fibrosis, especially atrial fibrosis, and previous studies report increased levels of these two biomarkers in patients with AF and coexisting cardiovascular disease or baseline before onset of AF [2, 5, 6, 9, 10, 22, 36]. This study found similar levels of these two biomarkers between the AF patients and healthy controls (Table 2), showing that TGF-β1 and PIIINP might not play an important role in the early stage of AF. However, both biomarkers were found to be significantly associated with LAV, and TGF-β1 was also significantly associated with LV peak systolic circumferential strain and strain rate, suggesting that TGF-β1 and PIIINP might play a minor role, relative to the roles of CRP and sST2, in the early stage of AF. Several mechanisms have been suggested for the different effects of TGF-β1 in the atria versus the ventricle; TGF-β1 up-regulation was more pronounced in the atria than in the ventricle [2]. A recent study showed that another fibrosis biomarker, type I collagen C terminal telopeptide has significant association with CMR-measured LV myocardial abnormality [37]. Further studies are needed to investigate the effects of these fibrotic biomarkers on the atrial and ventricular morphology and function in AF patients.

CMR-measured LV myocardium abnormality in AF patients without coexisting cardiovascular disease has rarely been reported in the literature. Using phosphorus-31 magnetic resonance spectroscopy, Wijesurendra et al. found that patients with AF and no apparent cardiovascular disease showed a reduced myocardial energetics (PCr/ATP, AF vs. controls: 1.81 ± 0.35 vs. 2.05 ± 0.29; P < 0.01) [13]. Ling et al. found that the AF patients without coexisting cardiovascular disease, a subgroup of the study population, showed significantly lower LV post-contrast T1 (AF vs. controls: 430 ± 96 ms vs. 518 ± 92 ms; P < 0.01) [12]. Using native T1 mapping, we observed a significantly higher LV pre-contrast T1 in the AF patient (Table 3). All these three studies suggested a LV myocardial abnormality in AF patients without coexisting cardiovascular disease. However, in comparison to the correlations of hs-CRP and sST2 with LAV and three LV strain indices, the correlation strengths of the native T1 with hs-CRP and sST2 were the weakest ones (Table 4). Nevertheless, the elevated LV native T1 time likely reflects fibrosis [12], which may be associated with increased myocardial stiffness/diastolic dysfunction, and the observed moderate associations between LV native T1 time and these two biomarkers raise a possibility that myocardial stiffness and inflammation may be involved in the process of myocardial fibrosis. Further studies are warranted to investigate this process. In addition, the native T1 change between the AF patients and healthy controls is relatively small (i.e., 2.3% from 1251 to 1280 ms). These results regarding the LV native T1 may reflect a subtle and diffuse change in the early stage of AF.

## Limitations

The sample size of the present study is small. Due to technical limitations on our site, we did not perform atrial LGE scans to examine the existence of atrial LGE lesions and its association with these circulating biomarkers. We only have 23 patients who underwent pulmonary vein isolation after CMR examination, and, due to these limited cases, we did not repeat the CMR examination and circulating biomarkers test after therapy for observing the impact of catheter ablation on circulating biomarkers and LV myocardium. Finally, we did not apply T2 mapping and post-contrast T1 mapping, and therefore the LV T2 value and extracellular volume fraction could not be provided.

## Conclusions

Both LA and LV abnormalities detected by CMR are associated with inflammation and cardiac remodeling biomarkers. CMR is a promising method for quantitatively assessing subtle LV myocardial impairments in patients with paroxysmal AF and no coexistent cardiovascular disease. CMR revealed abnormalities in LV contractility and tissue characterization prior to LV structural changes. This LV myocardial impairment analysis may provide insight in assessing the LV performance over time in AF patients.

## Supplementary Information

**Additional file 1: Table S1.** Inter- and intra-observer reproducibility measured with the intraclass correlation coefficient (ICC) for the CMR parameters.

## Data Availability

The datasets used and/or analyzed during the current study are available from the corresponding author on reasonable request.

## Abbreviations

ε_s_: Peak systolic circumferential strain
AF: Atrial fibrillation
BW: Band width
CMR: Cardiovascular magnetic resonance
CO: Cardiac output
CRP: C-reactive protein
DCS: Diastolic circumferential strain
FOV: Field-of-view
hs-CRP: High sensitivity C-reactive protein
LAV: Left atrial volume
LGE: Late gadolinium enhancement
LV: Left ventricle/left ventricular
LVEDV: Left ventricular end-diastolic volume
LVEF: Left ventricular ejection fraction
LVESV: Left ventricular end-systolic volume
MOLLI: Modified look-locker inversion recovery
PIIINP: Collagen III N-terminal propeptide
SCS: Systolic circumferential strain
SR: Strain rate
SRe: Early diastolic circumferential strain rate
SRs: Peak systolic circumferential strain
sST2: Soluble suppression of tumorigenicity
ST2: Suppression of tumorigenicity 2
SV: Stroke volume
TE: Echo time
TGF-β1: Transforming growth factor beta-1
TR: Repetition time
