# Supplementary material for: Cardiovascular magnetic resonance-determined left ventricular myocardium impairment is associated with C-reactive protein and ST2 in patients with paroxysmal atrial fibrillation
Source: J Cardiovasc Magn Reson. 2021 Mar 22;23:30. doi: 10.1186/s12968-021-00732-5 (PMC7983280; doi:10.1186/s12968-021-00732-5)
Supplement: Supplementary file 1 — Additional file 1: Table S1. Inter- and intra-observer reproducibility measured with the intraclass correlation coefficient (ICC) for the CMR parameters. [file 12968_2021_732_MOESM1_ESM.docx]

Table S1. Inter- and intra-observer reproducibility measured with the intraclass correlation coefficient (ICC) for the CMR parameters.

|  | Intra-observer | | Inter-observer | |
| --- | --- | --- | --- | --- |
|  | ICC | 95% CI | ICC | 95% CI |
| LAV | 0.93 | 0.85-0.96 | 0.89 | 0.80-0.95 |
| LV EF | 0.91 | 0.82-0.95 | 0.88 | 0.73-0.91 |
| LV EDV | 0.95 | 0.89-0.98 | 0.93 | 0.83-0.97 |
| LV ESV | 0.95 | 0.87-0.98 | 0.91 | 0.79-0.96 |
| LV SV | 0.92 | 0.83-0.96 | 0.89 | 0.77-0.94 |
| LV mass | 0.93 | 0.83-0.97 | 0.91 | 0.79-0.97 |
| LV peak SCS | 0.85 | 0.83-0.92 | 0.81 | 0.76-0.87 |
| LV peak SCS rate | 0.87 | 0.80-0.91 | 0.83 | 0.78-0.89 |
| LV early DCS rate | 0.91 | 0.84-0.95 | 0.88 | 0.81-0.92 |
| LV native T1 time | 0.94 | 0.86-0.98 | 0.91 | 0.79-0.96 |

CMR: cardiovascular magnetic resonance; CI: confidence interval; LAV: left atrium volume; EF: ejection fraction; EDV: end-diastolic volume; ESV: end-systolic volume; LV: left ventricle/left ventricular; SV: stroke volume; SCS: systolic circumferential strain; and DCS: diastolic circumferential strain.
